# CCL5 regulation of mucosal chlamydial immunity and infection

**DOI:** 10.1186/1471-2180-8-136

**Published:** 2008-08-13

**Authors:** Senthilkumar K Sakthivel, Udai P Singh, Shailesh Singh, Dennis D Taub, Joseph U Igietseme, James W Lillard

**Affiliations:** 1 Emory University School of Medicine, Department of Pathology, Atlanta GA, USA; 2 University of South Carolina School of Medicine, Department of Pathology, Microbiology, & Immunology, Columbia, SC, USA; 3 University of Louisville School of Medicine, Brown Cancer Center, Department of Microbiology & Immunology, Louisville, KY, USA; 4 National Institute of Aging, Gerontology Research Center, Laboratory of Immunology Baltimore, MD, USA; 5 National Center for Infectious Diseases, Centers for Disease Control & Prevention (CDC), Atlanta, GA, USA; 6 Morehouse School of Medicine, Department of Microbiology, Biochemistry, & Immunology, Atlanta, GA, USA

## Abstract

**Background:**

Following genital chlamydial infection, an early T helper type 1 (Th1)-associated immune response precedes the activation and recruitment of specific Th1 cells bearing distinct chemokine receptors, subsequently leading to the clearance of *Chlamydia*. We have shown that CCR5, a receptor for CCL5, is crucial for protective chlamydial immunity. Our laboratory and others have also demonstrated that CCL5 deficiencies found in man and animals can increase the susceptibility and progression of infectious diseases by modulating mucosal immunity. These findings suggest the CCR5-CCL5 axis is necessary for optimal chlamydial immunity. We hypothesized CCL5 is required for protective humoral and cellular immunity against *Chlamydia*.

**Results:**

The present study revealed that CCR5 and CCL5 mRNAs are elevated in the spleen, iliac lymph nodes (ILNs), and genital mucosa following *Chlamydia muriduram *challenge. Antibody (Ab)-mediated inhibition of CCL5 during genital chlamydial infection suppressed humoral and Th1 > Th2 cellular responses by splenic-, ILN-, and genital mucosa-derived lymphocytes. Antigen (Ag)-specific proliferative responses of CD4^+ ^T cells from spleen, ILNs, and genital organs also declined after CCL5 inhibition.

**Conclusion:**

The suppression of these responses correlated with delayed clearance of *C. muriduram*, which indicate chlamydial immunity is mediated by Th1 immune responses driven in part by CCL5. Taken together with other studies, the data show that CCL5 mediates the temporal recruitment and activation of leukocytes to mitigate chlamydial infection through enhancing adaptive mucosal humoral and cellular immunity.

## Background

*Chlamydia trachomatis *is the most common sexually transmitted bacterial pathogen in the United States; infection results in devastating sequelae, including pelvic inflammatory disease and infertility. Animal models and clinical studies of infected patients have indicated that protective chlamydial immunity is primarily mediated by Th1 responses [1-3]. The induction of such immunity involves rapid recruitment and activation of certain effector immune cells, specifically Th1 cells and dendritic cells (DCs) into the local genital mucosa to clear the infection, arrest ascending disease, and prevent major complications [1,4-7]. In addition, certain complimentary B cell functions – principally Ab-mediated enhancement of Ag presentation – lead to activation of Ag-specific Th1 cells dependent in part by Fc-receptor-mediated events [1,8]. Thus, both cell-mediated and humoral immune responses are required for long-term protection against *Chlamydia*.

With the major elements of protective anti-*Chlamydia *immunity defined, a number of candidate vaccines have been described [1]. Chemokines have emerged as important factors and possible mucosal adjuvants that function in lymphocyte activation and recruitment [9-11]. Indeed, a qualitative relationship exists between the class of chemokines secreted following infection, the type of immune response (cellular or humoral immunity) elicited, and the fate of the host following infection [12-15]. The profile of chemokine expression serves as a reliable indicator of immune response type (i.e., Th1 *vs*. Th2). In this respect, the CCL5-CCR5 axis has been demonstrated to be preferentially involved in the activation and function of Thl cells [10,16,17].

CCL5 is secreted by epithelial cells, macrophages, fibroblasts, platelets, and activated T cells [18]. This CC chemokine is known to regulate T cell differentiation and polarize Th1 >> Th2 subtypes [10,13,18,19] as well as numerous physiological functions of leukocytes including migration. Polymorphisms in CCR5 and CCL5 modulate immune responses as well as susceptibility and progression to HIV-1 and AIDS, respectively [20,21]. We also showed that many of the deleterious complications of genital chlamydial infection, due to Th1-mediated inflammation, are not present in individuals with the *ccr5Δ32 *mutation or in CCR5-deficient mice [22]. CCR5 expression following genital chlamydial infection is followed by an early Th1-like response that precedes activation and mucosal recruitment of Ag-specific Th1 cells necessary for clearance of *Chlamydia *[23]. These findings indicate that CCL5 might be important for inducing protective immunity against *Chlamydia*. However, it is not certain what affect CCL5 deficiency would have on chlamydial disease. We tested the hypothesis that CCL5 is essential for inducing adaptive mucosal immunity against *Chlamydia *by Ab inhibition using a reliable mouse model of genital chlamydial infection. Results revealed CCL5 supports the induction of Th1 cytokine and immunoglobulin IgG2a as well as IgA responses against *Chlamydia*.

## Results

### Expression of chemokines after genital Chlamydia infection

**Figure 1:**
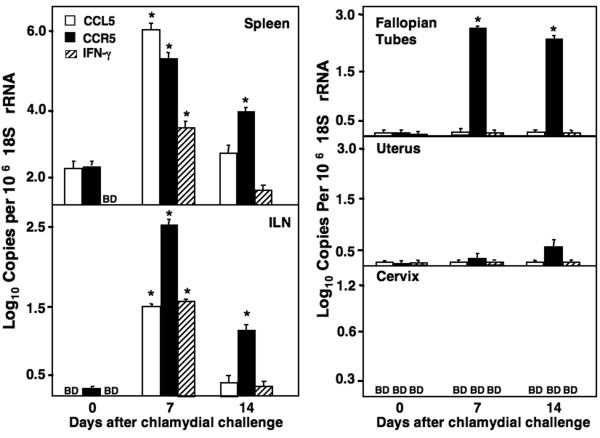
**CCL5, CCR5, and IFN-γ mRNA expression during *Chlamydia *infections**. Groups of female BALB/c mice challenged with *C. muridarum *and total RNA was isolated from lymphocytes isolated from the spleen, ILNs, fallopian tubes, uterus, and cervix of each mouse, under sterile conditions before or 7 and 14, 21 and 42 days after challenge. The levels of CCL5, CCR5 and IFN-γ mRNA expression were ascertained after RT-PCR analysis. Log_10 _copies of transcripts were expressed relative to the actual copies of 18S rRNA ± SEM. Experiments were repeated 3 times to yield 15 mice per group. Asterisks (*) indicate significant differences (*p *< 0.01) between tissues from naïve mice.

CCL5, CCR5, and IFN-γ mRNAs were measured by quantitative RT-PCR analysis after genital chlamydial infection. A significant increase in CCR5, CCL5, and IFNγ gene expression in the spleen and ILN was observed 7 days after genital infection when compared with levels before infection (Figure 1). These mRNA levels modestly declined at inductive sites 14 days after infection. CCR5, but not CCL5 or IFN-γ mRNA expression by fallopian tube-, uterus-, and cervix- derived lymphocytes were considerably higher than levels before infection. Indeed, CCR5 mRNA expression by fallopian tube lymphocytes was significantly higher 7 and 14 days post infection. These data suggest that increases in CCL5, CCR5, and IFN-γ mRNA expression during early stages of infection at inductive sites (i.e., spleen and ILN) preceded CCR5 expression at effectors sites (i.e., fallopian tube(s), uterus, and cervix). The pattern of CCL5 and CCR5 as well as IFN-γ illustrates how this chemokine axis coincides with both the innate (recognition phase, 0 to 7 days) and adaptive (activation, effector, and decline/homeostasis phases) immune responses to this pathogen and associated inflammation.

### Proliferative responses of *C. muridarum *genital infection modulated by CCL5 inhibition

**Figure 2:**
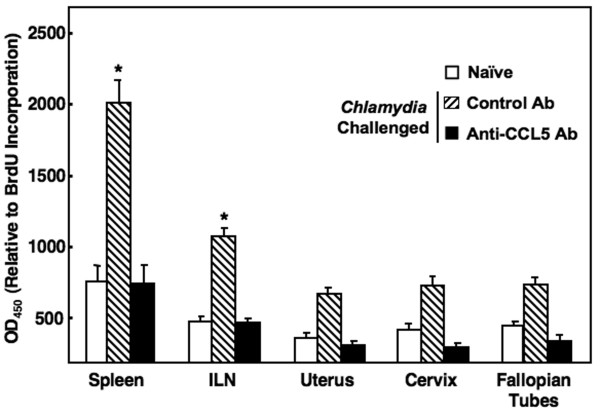
**Chlamydia-specific CD4^+ ^T cell proliferation**. Groups of naïve or female BALB/c mice challenged with *C. muridarum *and received 100 μl of control Ab or anti-CCL5 Ab solution every 3 days. Following sacrifice 42 days after challenge, spleen-, ILN-, fallopian tubes-, uterus-, and cervix-derived CD4^+ ^T cells were purified and cultured at a density of 5 × 10^6 ^cells/ml with 10^6 ^cells/ml of γ-irradiated feeder splenocytes for 3 days. Proliferation of CD4^+ ^T cells was measured by BrdU incorporation. The data presented are the mean OD_450 _for proliferative ± SEM of quadruplicate cultures. Experiments were repeated 3 times to yield 15 mice per group. Asterisks (*) indicate statistically significant differences (*p *< 0.01) between untreated, control Ab-treated mice and mice treated with anti-CCL5 Ab.

We next characterized *C. muridarum*-specific proliferative responses of T helper cells isolated from the spleen, ILNs, fallopian tube(s), uterus, and cervix, 42 days after challenge. CD4^+ ^T cells isolated from these mucosal and systemic immune compartments of *C. muridarum*-infected and control Ab-treated mice exhibited marked increases in Ag-specific proliferative responses compared to disease-free (uninfected) or *C. muridarum*-infected and anti-CCL5 Ab-treated mice (Figure 2). Notably, systemic and mucosal inductive sites of the spleen and ILN, respectively, contained Ag-specific CD4^+ ^T cells that significantly proliferated after *C. muridarum *re-stimulation. These results suggest that CCL5 is required for optimal generation of *Chlamydia*-specific CD4^+ ^T cells that proliferate after Ag recognition.

### CCL5 modulation of Chlamydia-specific humoral responses

**Figure 3:**
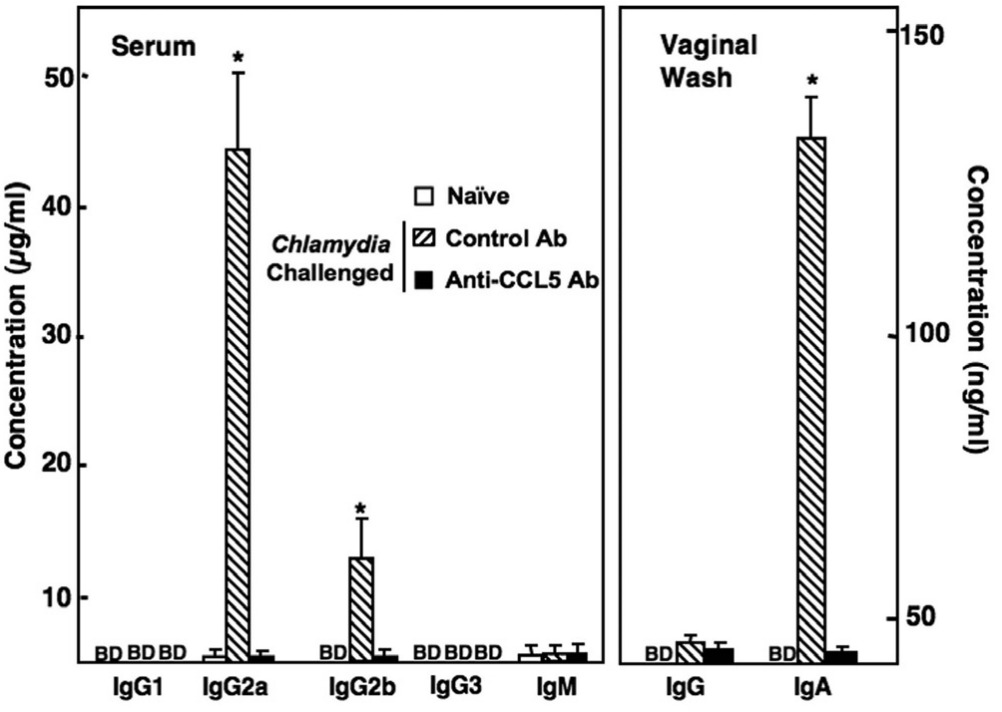
**Serum and vaginal Ab responses of mice infected with genital *Chlamydia***. Groups of naïve or female BALB/c mice challenged with *C. muridarum *and received 100 μl of control Ab or anti-CCL5 Ab solution every 3 days. Following sacrifice 42 days after challenge, *Chlamydia*-specific serum and vaginal Ab responses of mice were determined by ELISA that was capable of detecting > 20 pg/ml of IgM, IgG, IgA, and IgG subclass Abs. The data presented are the mean concentration of IgG1, IgG2a, IgG2b, IgG3, IgM, IgG, or IgA ± SEM of three separate experiments. Experiments were repeated 3 times to yield 15 mice per group. Asterisks (*) indicate statistically significant differences (*p *< 0.01) between untreated and control Ab- or anti-CCL5 Ab-treated mice.

To test the role of CCL5 in Th1-biased humoral responses to genital infection, we measured *Chlamydia*-specific IgG1, IgG2a, IgG2b, IgG3 and IgM Abs in sera as well as Ag-specific IgA and IgG Abs in vaginal washes. Infected mice that received control Ab displayed significantly higher levels of *Chlamydia*-specific serum IgG2a, followed by IgG2b responses than compared to similar mice that received anti-CCL5 Ab or mock-infected (i.e., naïve) mice (Figure 3). Analysis of vaginal secretions revealed a significant increase in Ag-specific IgA Ab responses in *C. muridarum*-challenged mice that received control Ab when compared with similar mice that received anti-CCL5 Ab or uninfected animals. These results indicate *C. muridarum *infection enhanced Th1-biased humoral responses and CCL5 blockade attenuated *Chlamydia*-specific IgG2a serum responses as well as vaginal IgA responses.

### T helper cytokine responses and *C. muridarum *shedding

**Figure 4:**
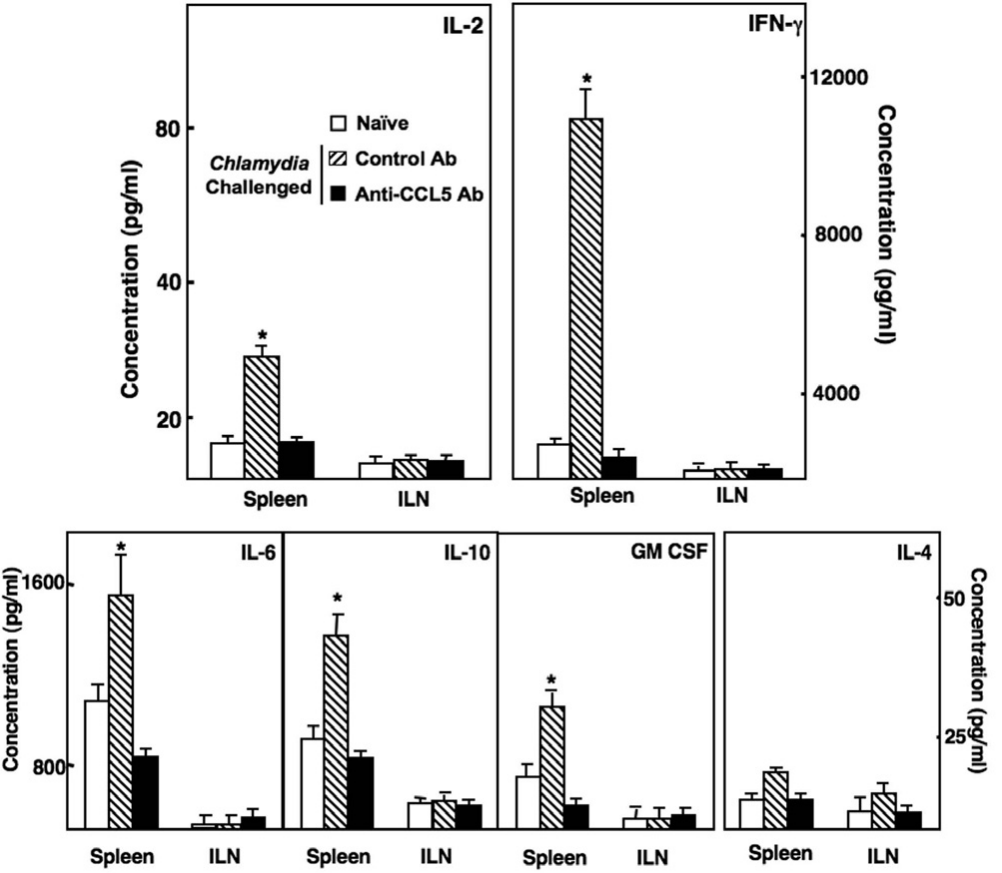
**T helper cytokine secretion by CD4^+ ^T cells from *Chlamydia*-infected mice**. Groups of naïve or female BALB/c mice challenged with *C. muridarum *and received 100 μl of control Ab or anti-CCL5 Ab solution every 3 days. Following sacrifice 42 days after challenge, spleen- and ILN-derived CD4^+ ^T cells from these mice were purified and cultured at a density of 5 × 10^6 ^cells/ml with 10^6 ^cells/ml of γ-irradiated feeder splenocytes for 3 days. Cytokines present in cultured supernatants were determined by ELISA that was capable of detecting < 10 pg of IL-2, IFN-γ, IL-4, IL-6, IL-10, or GM-CSF. The data presented are the mean cytokine (pg/ml) ± SEM of quadruplicate cultures. Asterisks (*) indicate statistically significant differences (*p *< 0.01) between untreated and control Ab- or anti-CCL5 Ab-treated mice.

**Figure 5:**
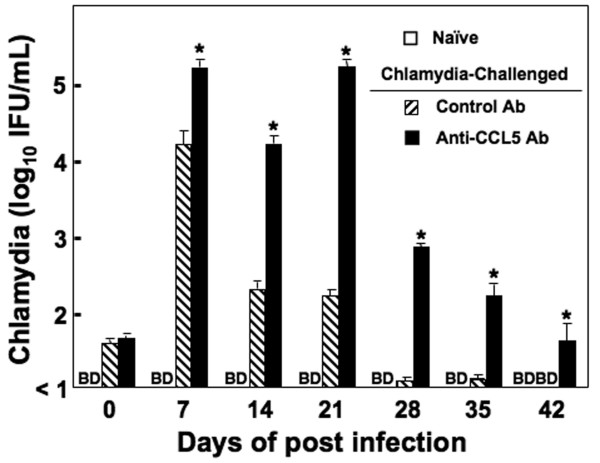
**Changes in *Chlamydia*-shedding**. Mice treated with control Ab or anti-CCL5 Ab were infected with *C. muridarum*. The status of infection was monitored for live organism shedding by culturing periodic cervico-vaginal swabs from individual mice every week for 42 days after challenge. *Chlamydia *present in swabs were detected by infecting McCoy cells with vaginal swab rinses and staining infected monolayer with FITC-labeled, genus-specific, anti-*Chlamydia *Abs to count inclusion bodies (> 10 IFUs/ml) by direct immuno-fluorescence. BD indicates the presence of *Chlamydiae *was below this level of detection. Experiments were repeated 3 times to yield 15 mice per group. Asterisks (*) indicate statistically significant differences (*p *< 0.01) between untreated and control Ab- or anti-CCL5 Ab-treated mice.

Genital *Chlamydia *infection up-regulated Ab responses as well as splenic and ILN CD4^+ ^T cell proliferative responses. We next examined whether these effects were mediated in part through T helper cytokine responses. IL-2 secreted by Ag-stimulated splenic CD4^+ ^T cells from infected mice that received control Ab was significantly higher than levels from cells isolated from similar mice that received anti-CCL5 Ab or uninfected mice (Figure 4). The secretion of IFN-γ from splenic, but not ILN, CD4^+ ^T cells from *C. muridarum*-infected and control Ab-treated were considerably higher than T helper cells isolated from similar mice treated with anti-CCL5 Ab or uninfected animals. Chlamydial infection of mice lead to the development of CD4^+ ^T cells that significantly secreted IL-6, IL-10, and GM-CSF, but not IL-4, in response to *C. muridarum *re-stimulation. Anti-CCL5 Ab-treated mice infected with *C. muridarum *resulted in T helper cells with reduced IL-6, IL-10, and GM-CSF secretion following Ag re-stimulation when compared to CD4^+ ^T cells from similar mice treated with control Ab; however, the secretion pattern of Th2 cytokine responses by CD4^+ ^T cells isolated from ILNs did not significantly change. These results indicate that *C. muridarum *infection invoked splenic, but not ILNs, *Chlamydia-*specific CD4^+ ^T cells that secreted Th1 cytokines as well as IL-6, IL-10, and GM-CSF, which were reduced by CCL5 blockade. The extent of chlamydial infection, determined by C. muridarum detected in cervico-vaginal swabs, was significantly higher in mice receiving anti-CCL5 Ab treatment than compared to control mice (Figure 5). However, all mice resolved chlamydial infection in < 90 days.

## Discussion

Th1-mediated immune responses are essential to control the chlamydial infection [22]. It has been shown in previous studies that CCL3, CCL4 and CCL5 enhance adaptive immunity through Th1 cytokine and co-stimulatory molecule modulation [10,11,15,16]. In this study, we demonstrate some of the cellular and molecular mechanisms of CCL5-mediated chlamydial immunity. Importantly, IFN-γ mRNA was not significantly elevated 14 days after *C. muridarum *infection. IL-12p40 mRNA expression coincided with CCL5, but typically preceded IFN-γ mRNA expression (data not shown). Indeed, early genital clearance of *Chlamydia *has been shown to occur in an IL-12-dependent and IFN-γ-independent fashion [24]; so, further studies will be required to dissect the roles of IL-12 and CCL5 in *Chlamydia *clearance and immunity. The present study shows that 7 days after genital *Chlamydia *infection, CCL5, CCR5, and IFNγ mRNA levels were elevated in inductive sites, while CCR5 mRNA expression was higher in the fallopian tubes than in the uterus and cervix. To this end, similar yet differential chemokine expression patterns have been reported within anatomically distinct regions [25].

CD4^+ ^Th1 cells (and associated cytokines, chemokines, etc.) are critical elements in the immune response, stimulated by an ascending *C. trachomatis *infection in the female genital tract [26]. Indeed, studies of chlamydial infection in knockout mice support the importance of class II MHC, CD4, IL-12, IFN-γ, and IFN-γ receptor for chlamydial immunity [27-30]. Our data suggest that CCL5 interactions are comparably important and mediate the temporal recruitment and activation of T cells to mitigate chlamydial infection through protective mucosal adaptive immunity by enhancing Th1 **>> **Th2 humoral and cellular immune responses.

CCL3 and CCL4 are also CCR5 ligands, but these chemokines were not remarkably elevated compared to CCL5, which was the most abundant CCR5 ligand expressed during the early stages (i.e., < 7 days) of chlamydial infection. CCL5 is an important factor in the homing of lymphocytes that express CCR5, CCR4, CCR3 and CCR1 [31-33]. It was also demonstrated from our earlier studies that CCL5 can increase the proliferation and activation of Ag stimulated T lymphocytes [10]. These findings coincide with marked increases in CCL5 and IFN-γ production by CD4^+ ^T cells during chlamydial infection. It is also plausible that CCL5 blockade might reduce the ability of innate immune cells (e.g., NK cells, macrophages, etc.) to respond to *C. muriduram *challenge. Previously, we demonstrated that CCL5 inhibition decreased the numbers of NK1.1^+ ^and CD11b^+ ^leukocytes at mucosal effector sites following pneumococcal challenge [34]. However, T cells were the primary lymphocyte-subtype that were reduced following CCL5 blockade during the hyper cellular response to *Streptococcus pneumoniae *in mice.

Genital *Chlamydia *infection of mice enhanced Ag-specific Abs in serum and vaginal secretions as well as proliferative cytokine responses by CD4^+ ^T cells isolated from systemic and mucosal compartments. High levels of IgA in cervical secretions in infected women correlate with low numbers of *C. trachomatis *shedding and B cells are required to eliminate *Chlamydia *in a secondary infection [35]**. **We have previously shown that CCL5 induces Ag-specific titers of IgG2a, followed by IgG2b, IgG3, and IgG1 [10], CCL3 and CCL4 enhance Ag-specific IgG1 and IgG2b responses [11]. In this study, *Chlamydia *infection enhanced IgG2a Ab responses, which were reduced after CCL5 inhibition. This highlights the importance of this chemokine in Th1-associated Ab responses against *Chlamydia*.

The precise cytokine signals required for S-IgA production are not completely understood and studies show both Th1-and Th2-type cell-derived cytokines are important for S-IgA production [36-38]. We have previously shown that chemokines like XCL1 and CCL5 induce IgA production [9,10]. The sharp IgA Ab response generated by *Chlamydia-*infected mice also correlated with the predominant Th1 >> Th2 cytokine response induced by this infection, which were reduced by CCL5 blockade. These results suggest that CCL5 is required in part for optimal *Chlamydia*-specific IgA Ab responses during *C. muridarum *infection.

Mucosal, but not serum, IgA Abs were selectively elevated after *C. muridarum *challenge, presumbably due to the compartmentalized common mucosal immunity system. In particular, IgA Ab-secreting cells predominantly populate the lamina propria of the mucosa. In contrast, *Chlamydia*-specific IgG Abs were not detected in vaginal secretions. This confirms other studies that show vaginal *Chlamydia*-specific IgG Abs are not present at high levels (relative to IgA) in vaginal washes after *C. muridarum *challenge [39,40]. However, intranasal, subcutaneous or transcutaneous immunization using mucosal adjuvants can induce vaginal wash Ag-specific IgG Ab titers that are comparable to those of IgA [9,41-45].

IFN-γ production is often associated with IgG2a production [46] and may account for the systemic humoral responses against *Chlamydia *following vaginal challenge. In the present study, *Chlamydia *infection induced a profound IgG2a Ab response compared to other IgG subclass Abs. The analysis of mucosal and systemic responses revealed a bias toward Th1 >> Th2 type of responses. While little is known regarding Ag-specific CD4^+ ^T cell (IL-4 and GM-CSF) secretory responses during *Chlamydia *infection, we show that these T helper cytokines were increased during chlamydial infection. CCL5 blockade diminished these cellular responses along with *C. muridarum*-specific CD4^+ ^T cell secretion of IFN-γ and IL-2.

Contradictory studies demonstrated the ability of CCL5 to promote both Th1-and Th2-type responses. It was reported that anti-CCL5 Ab treatment in mice decreased mycobacterial-inducible Th2-type lesions while increasing schistosomal-inducible Th2-type granulomas [47]. However, studies from our laboratory suggest CCL5 enhances mucosal and systemic humoral responses through help provided by Th1-type cytokines and select Th2-type cytokines, with CCL5 promoting Th1 and Th2 responses [10]. The results in the present study suggest that CCL5 is required for optimal Th1 cellular responses against and clearance of *Chlamydia*. These results corroborate our previous findings that CCR5-dependent mucosal immune responses are required for the efficient clearance of genital chlamydial infection, while functional CCR5 expression reduces infertility as a pathologic consequence of Th1-mediated inflammation associated with infection [22].

The *ccl5 *gene has a number of physiologically relevant single nucleotide polymorphisms that affect its function and expression. In particular, the In1.1T/C haplotype of *ccl5 *is highly prevalent (~37%) in African Americans, than compared to Americans of European origin (~0.3%), and results in significantly lower CCL5 expression [48]. This has been attributed to some of the health disparities between these ethic/racial groups; specifically, higher human immunodeficiency virus (HIV) susceptibility and faster progression to acquired immune deficiency syndrome (AIDS). Similar to the *ccr5Δ*32 polymorphism, the data in this study and others suggest diminished CCL5-CCR5 interactions could yield reduced fallopian tube scarring, but could also result in higher transmission/shedding of *Chlamydia *via decreased chlamydial clearance. It is plausible that a host with a modestly compromised innate immune system would on one hand – avoid infertility by mounting reduced Th1 responses and on the other – be unable to optimally clear a chlamydial infection. This would provide an evolutionary rationale for not only *ccr5Δ*32 and *In1.1T/C ccl5 *polymorphisms, but also the propagation and transmission of *Chlamydia*. No doubt, demonstration of this postulate will require additional and extensive studies.

## Conclusion

Understanding the cellular and molecular mechanisms that CCL5 uses to modulate mucosal immunity is essential to better understanding the pathogenesis of chlamydial infection. We tested the hypothesis that CCL5 is essential for inducing adaptive mucosal immunity against *Chlamydia*. We conclude that CCL5 supports the induction of Th1 cytokine and IgG2a Ab as well as IgA Ab responses against *Chlamydia*. The suppression of CCL5 correlated with delayed clearance of *C. muriduram *infection, which suggests chlamydial immunity is mediated by Th1 immune responses driven in part by CCL5.

## Methods

### *Chlamydia *stocks

Stocks of the *Chlamydia muridarum *(trachomatis agent for mice) were prepared by propagating elementary bodies (EBs) in McCoy cells and used to infect mice as previously described [49]. The stocks were tittered by infecting McCoy cells with varying dilutions of EBs. The infectious titer was expressed as inclusion-forming units per milliliter (IFU/ml).

### Anti-CCL5 antibody production

Murine anti-CCL5 was produced as previously described [34]. Briefly, rabbits were immunized with murine CCL5 (Pepro-Tech) and the hyper immune CCL5 antisera obtained was yielded titers of ~1:10^6 ^such that 10 μl of rabbit CCL5 antiserum neutralized 20 ng of CCL5. This antiserum was titrated by direct ELISA, and no cross-reactivity was detected when tested against other CCR5 ligands, chemokines and cytokines. Subsequently, antisera were heat-inactivated and purified using an IgG isotype-specific protein A column (Pierce). Anti-CCL5 Ab titers were adjusted to 1 : 4 × 10^5 ^(i.e., 50× dilution) in PBS (Ab solution) for CCL5 blocking experiments. Similarly prepared normal or preimmune rabbit serum was used to generate the control Ab solution.

### Animal infection, Ab treatment, and analysis of disease course

Female BALB/c mice, ages 6 to 8 weeks, were purchased from Jackson Laboratory and used to establish a colony at the Morehouse School of Medicine animal facility. The animals were housed and maintained in isolator cages under specific-pathogen-free housing conditions. The guidelines proposed by the Committee for the Care of Laboratory Animal Resources Commission of Life Sciences-National Research Council were followed to minimize animal pain and distress. These studies were approved by the Morehouse School of Medicine Institutional Animal Care and Use Committee (IACUC). Seven days prior to infection, mice received 2.5 mg of medroxy-progesterone acetate (Depo-Provera; The Upjohn Co.) by subcutaneous route in 100 μl of PBS [50]. Groups were intravaginally infected with 5 × 10^4 ^IFU of *C. muridarum *under phenobarbital anesthesia, whereas uninfected control mice received only phosphate buffered saline (PBS). Mice (15 mice per group) received 100 μl of either control or anti-CCL5 Abs by intraperitoneal injection, 24 hrs before chlamydial infection and every 72 hrs thereafter. The extent of unresolved chlamydial infection was monitored by taking cervico-vaginal swabs from individual mice every week until 42 days after the challenge [22]. Briefly,*Chlamydia *present in swabs were detected by infecting McCoy cells vaginal/swab rinses and staining infected monolayer with fluorescein isothiocyanate (FITC)-labeled, genus-specific, anti-*Chlamydia *Abs (Kallestad Diagnostics) to verify inclusion bodies (> 10 IFUs/ml) by direct immuno-fluorescence [49].

### Sample, tissue, and cell collection

Vaginal cavities were rinsed three times with 50 μl of PBS to obtain mucosal secretions. Blood collected by retro-orbital bleeding and serum was separated following centrifugation. Serum and mucosal secretions were collected on 0, 7, 14, and 42 days post challenge and analyzed by ELISA. Following sacrifice by CO_2 _inhalation, spleen, ILN, fallopian tube(s), uterus, and cervix tissues were aseptically removed and single-cell suspensions were prepared by passing tissues through a sterile wire screen to quantify mRNA expression and T helper responses. Reproductive tract tissues were further disrupted to generate single cell suspensions by stirring in collagenase type IV (Sigma) in RPMI 1640 (collagenase solution) at 37°C for 30 min. Lymphocytes were further purified using a discontinuous Percoll (Pharmacia) gradient, collected at the 40/75% interface. CD4^+ ^T cells were enriched using Mouse CD4 Cellect^® ^plus columns according to manufacturer's protocol (Biotex Laboratories). Cell suspensions were washed twice in RPMI 1640 and lymphocytes were maintained in medium supplemented with 10 ml/L of nonessential amino acids (Mediatech), 1 mM sodium pyruvate (Sigma), 10 mM HEPES (Mediatech), 100 U/ml penicillin, 100 μg/ml streptomycin, 40 μg/ml gentamycin (Elkins-Sinn), 50 μM mercaptoethanol (Sigma), and 10% fetal calf serum (FCS) (Atlanta Biologicals).

### Chlamydia-specific T helper cell responses

Purified CD4^+ ^T cells were cultured at a density of 5 × 10^6 ^cells/ml with 10^6 ^cells/ml γ-irradiated (3,000 rads) naïve splenic feeder cells in complete medium in the presence or absence of 10 μg/ml of UV-inactivated *C. muridarum *inclusion bodies (IBs) as Ag at 37°C in 5% CO_2_. After 3 days of culture, cells were pulsed with BrdU labeling solution and incorporation was detected by ELISA (Roche Molecular Biochemical). Similarly, cell culture supernatants were collected 3 days after culture and assayed for cytokine secretion by Luminex assay [34].

### Anti-Chlamydia Ab detection in serum and vaginal washes

*Chlamydia*-specific serum IgG subclass antibodies and vaginal wash IgA levels were quantified 6 weeks after challenge by ELISA [10]. Briefly, 96-well Falcon ELISA plates (Fisher Scientific) were coated with 100 μl of anti-IgG or IgA Ab (BD-PharMingen) or 10 μg/ml of UV-inactivated *C. muridarum *IBs in PBS O/N at 4°C and blocked with 10% FCS in PBS for 2 hrs at RT. IgM, IgG subclasses or IgA standards, and experimental samples were serially added after diluted with PBS. After O/N incubation at 4°C and three washes using PBS containing 0.05% Tween 20 (PBS-T), Ag-specific titers of IgM, IgG, IgA, or IgG subclass Abs were determined following the addition of biotinylated detection Abs (BD-PharMingen). After incubation and wash steps, anti-biotin HRP Ab (Vector Laboratories Inc., Burlingame, CA) in PBS-T was added to detection wells and incubated for 1 hr at RT. Following incubation, all plates were washed 6 times and the color reaction was developed by adding 100 μl of 1.1 mM 2,2'-azino-bis(3)-ethylbenzthiazoline-6-sulfonic acid (Sigma) in 0.1 M citrate-phosphate buffer (pH 4.2) containing 0.01% H_2_O_2 _(ABTS solution).

### Cytokine detection

The presence of the following T helper cell-derived cytokines in culture supernatants was determined by Beadlyte™ mouse multi-cytokine detection system kit (BioRad): interleukin (IL)-2, IL-4, IL-6, IL-10, granulocyte monocyte cell stimulating factor (GM-CSF), and interferon (IFN)-γ. Filter-bottom ELISA plates (BioRad) were rinsed with 100 μl of Bioplex assay buffer and the buffer was removed using a Millipore™ multiscreen separation vacuum manifold system set at 5 mm Hg. Analyte beads in assay buffer were added into wells, followed by 50 μl of serum or standard solution and incubated for 30 mins at RT with continuous shaking (at setting #3) using a Lab-Line™ Instrument Titer Plate Shaker (Melrose, IL). The filter-bottom plates were washed as before and the buffer was removed using a Millipore™ multiscreen separation vacuum manifold system. Subsequently, 50 μl of anti-mouse IL-2, IL-4, IL-6, IL-10, GM-CSF, or IFN-γ Ab-biotin reporter solution was added in each well and the plates were incubated with continuous shaking for 30 mins followed by centrifugation and washing. Next, 50 μl of streptavidin-phycoerytherin (PE) solution was added and the plates were incubated with continuous shaking for 10 mins at RT. 125 μl of Bio-plex assay buffer was added and Beadlyte™ readings were measured using a Luminex™ System and calculated using Bio-plex™ software (Bio-Rad). The cytokine Beadlyte™ assays were capable of detecting > 5 pg/ml for each analyte.

### RNA isolation and gene expression analysis

Total RNA from the spleen, ILNs, fallopian tube, uterus, and cervix leukocytes was isolated from mouse treated with anti-CCL5 or control Ab using Tri-reagent™ (Molecular Research Center, Cincinnati, OH). Potential genomic DNA contamination was removed from these samples by treatment with RNase-free DNase (Invitrogen) for 15 mins at 37°C. RNA was then precipitated and re-suspended in RNA secure (Ambion). cDNA was generated by reverse transcribing approximately 1.5 μg of total RNA using Taqman™ reverse transcription reagent (Applied Biosystems).

Mouse mRNA sequences of CCL5, CCR5, IFN-γ, and 18S rRNA were obtained from the NIH-NCBI gene bank database accession numbers NM03653, D83648, K00083, and X00686.1, respectively. These sequences were then used to design primers for real-time polymerase chain reaction (RT-PCR) analysis, which generated amplicons of 97, 100, 98, and 149 base pairs size, respectively, for CCL5 (sense-TCG TGT TTG TCA CTC GAA GG and antisense- GCT GAT GGC CTG ATT GTC TT), CCR5 (sense- CGA AAA CAC ATG GTC AAA CG and antisense- GGG AAG CGT ATA CAG GGT CA, IFN-γ (sense- ACT GGC AAA AGG ATG GTG AC and antisense- GTT CTC CTG TGG ATC GGG TA), and 18S rRNA (sense-GTA ACC CGT TGA ACC CAA TT and antisense- CAA TCC AAT CGG TAG TAG CG). Primers were designed using the primer 3 software program from Whitehead Institute at the Massachusetts Institute of Technology (MIT). Thermodynamic analysis of primers was conducted using the following computer programs: Primer Premier™ (Integrated DNA Technologies) and MIT Primer III (Boston, MA). The resulting primer sets were compared against the entire murine genome using the National Center for Biotechnology Information (NCBI) to confirm specificity and ensure that the primers flanked mRNA splicing regions. cDNA was generated as before and amplified with specific cDNA primers using SYBR^® ^Green PCR master mix reagents (Applied Biosystems). The copy number (> 10) of mRNA relative to 18S rRNA copies was evaluated by RT-PCR analysis using the BioRad Icycler and software (Hercules, CA).

### Statistics

Data were expressed as the mean ± standard error of mean (SEM), compared using a two-tailed student's *t*-test or an unpaired Mann Whitney *U*-test, and considered statistically significant if *p *< 0.01. When cytokine levels were below detection (BD) limit, they were recorded as one-half the lower detection limit (e.g., 5 pg/ml for IL-10) for statistical analysis.

## Abbreviations

Ab: antibody; ABTS: 2,2'-azino-bis(3)-ethylbenzthiazoline-6-sulfonic acid; Ag: antigen; AIDS: acquired immunodeficiency syndrome; BD: below detection; BrdU: bromodeoxyuridine; DAB: diaminobenzedine tetrahydrochloride; DC: dendritic cell; EB: elementary body; FCS: fetal calf serum; FITC: fluorescein isothiocyanate; GMCSF: granulocyte monocyte cell stimulating factor; HIV: human immunodeficiency virus; HRP: horse radish peroxidase; IB: inclusion body; IFN: interferon; IFU/ml: inclusion-forming units per milliliter; Ig: immunoglobulin; IL: interleukin; ILN: iliac lymph node; MHC: major histocompatibility complex; MIT: Massachusetts Institute of Technology; NCBI: National Center for Biotechnology Information; OD: optical density; O/N: over night; PBS: phosphate buffered saline; PBS-T: PBS Tween; PE: phycoerytherin; RPMI: Roswell Park Memorial Institute; RT: room temperature; RT-PCR: real-time polymerase chain reaction; SEM: Standard Error of Mean; TBS: tris-buffered saline; Th1: T helper type 1; TNF: tumor necrosis factor; UV: ultra violet.

## Authors' contributions

SKS and UPS carried-out all animal studies. SKS quantified serum and vaginal wash Ab levels as well as *C. muriduram *shedding. UPS isolated and measured mRNA levels as well as T cell cytokine secretion. DDT provided CCL5 and anti-CCL5 Ab as well as helped to draft the manuscript. JUI provided *C. muriduram *EBs and determined the corresponding titer. JWL as well as JUI conceived the study, participated in its design with all authors, coordinated and helped to draft the manuscript with the assistance of all authors. All authors read and approved the final manuscript. The authors declare that they have no competing interests.

## References

[B1] Igietseme JU, Black CM, Caldwell HD (2002). *Chlamydia *vaccines: strategies and status. Biodrugs.

[B2] Morrison RP, Caldwell HD (2002). Immunity to murine chlamydial genital infection. Infect Immun.

[B3] Loomis WP, Starnbach MN (2002). T cell responses to *Chlamydia trachomatis*. Curr Opin Microbiol.

[B4] Cain TK, Rank RG (1995). Local Th1-like responses are induced by intravaginal infection of mice with the mouse pneumonitis biovar of *Chlamydia trachomatis*. Infect Immun.

[B5] Igietseme JU, Ananaba GA, Candal DH, Lyn D, Black CM (1998). Immune control of Chlamydial growth in the human epithelial cell line RT4 involves multiple mechanisms that include nitric oxide induction, tryptophan catabolism and iron deprivation. Microbiol Immunol.

[B6] Stagg AJ, Tuffrey M, Woods C, Wunderink E, Knight SC (1998). Protection against ascending infection of the genital tract by *Chlamydia trachomatis *is associated with recruitment of major histocompatibility complex class II antigen-presenting cells into uterine tissue. Infect Immun.

[B7] Kelly KA, Walker JC, Jameel SH, Gray HL, Rank RG (2000). Differential regulation of CD4 lymphocyte recruitment between the upper and lower regions of the genital tract during *Chlamydia trachomatis *infection. Infect Immun.

[B8] Moore T, Ananaba GA, Bolier J, Bowers S, Belay T, Eko FO, Igietseme JU (2002). Fc receptor regulation of protective immunity against *Chlamydia trachomatis*. Immunol.

[B9] Lillard JW, Boyaka PN, Hedrick JA, Zlotnik A, McGhee JR (1999). Lymphotactin acts as an innate mucosal adjuvant. J Immunol.

[B10] Lillard JW, Boyaka PN, Taub DD, McGhee JR (2001). RANTES potentiates antigen-specific mucosal immune responses. J Immunol.

[B11] Lillard JW, Singh UP, Boyaka PN, Singh S, Taub DD, McGhee JR (2003). MIP-1alpha and MIP-1beta differentially mediate mucosal and systemic adaptive immunity. Blood.

[B12] Sallusto F, Lanzavecchia A, Mackay CR (1998). Chemokines and chemokine receptors in T-cell priming and Th1/Th2-mediated responses. Immunol Today.

[B13] Luther SA, Cyster JG (2001). Chemokines as regulators of T cell differentiation. Nature Immunology.

[B14] Darville T, Andrews CW, Sikes JD, Fraley PL, Braswell L, Rank RG (2001). Mouse strain-dependent chemokine regulation of the genital tract T helper cell type 1 immune response. Infect Immun.

[B15] Schrum S, Probst P, Fleischer B, Zipfel PF (1996). Synthesis of the CC-chemokines MIP-1alpha, MIP-1beta, and RANTES is associated with a type 1 immune response. J Immunol.

[B16] Bonecchi R, Bianchi G, Bordignon PP, D'ambrosio D, Lang R, Borsatti A, Sozzani S, Allavena P, Gray PA, Mantovani A, Sinigaglia F (1998). Differential expression of chemokine receptors and chemotactic responsiveness of type 1 T helper cells (Th1s) and Th2s. J Exp Med.

[B17] Sallusto F, Mackay CR, Lanzavecchia A (1997). Selective expression of the eotaxin receptor CCR3 by human T helper 2 cells. Science.

[B18] Appay V, Rowland-Jones SL (2001). RANTES: a versatile and controversial chemokine. Trends Immunol.

[B19] Makino Y, Cook DN, Smithies O, Hwang OY, Neilson EG, Turka LA, Sato H, Wells AD, Danoff TM (2002). Impaired T cell function in RANTES-deficient mice. Clinical Immunol.

[B20] Liu H, Chao D, Nakayama EE, Taguchi H, Goto M, Xin X, Takamatsu JK, Saito H, Ishikawa Y, Akaza T, Juji T, Takebe Y, Ohishi T, Fukutake K, Maruyama Y, Yashiki S, Sonoda S, Nakamura T, Nagai Y, Iwamoto A, Shioda T (1999). Polymorphism in RANTES chemokine promoter affects HIV-1 disease progression. Proc Natl Acad Sci USA.

[B21] McDermott DH, Beecroft MJ, Kleeberger CA, Al-Sharif FM, Ollier WE, Zimmerman PA, Boatin BA, Leitman SF, Detels R, Hajeer AH, Murphy PM (2000). Chemokine RANTES promoter polymorphism affects risk of both HIV infection and disease progression in the Multicenter AIDS Cohort Study. Aids.

[B22] Barr EL, Ouburg S, Igietseme JU, Morre SA, Okwandu E, Eko FO, Ifere G, Belay T, He Q, Lyn D, Nwankwo G, Lillard J, Black CM, Ananaba GA (2005). Host inflammatory response and development of complications of *Chlamydia trachomatis *genital infection in CCR5-deficient mice and subfertile women with the CCR5delta32 gene deletion. J Microbiol Immunol Infect.

[B23] Belay T, Eko FO, Ananaba GA, Bowers S, Moore T, Lyn D, Igietseme JU (2002). Chemokine and chemokine receptor dynamics during genital chlamydial infection. Infect Immun.

[B24] Perry LL, Feilzer K, Caldwell HD (1997). Immunity to *Chlamydia trachomatis *is mediated by T helper 1 cells through IFN-gamma-dependent and -independent pathways. J Immunol.

[B25] Maxion HK, Kelly KA (2002). Chemokine expression patterns differ within anatomically distinct regions of the genital tract during *Chlamydia trachomatis *infection. Infect Immun.

[B26] Johansson M, Schon K, Ward M, Lycke N (1997). Studies in knockout mice reveal that anti-chlamydial protection requires TH1 cells producing IFN-gamma: is this true for humans?. Scand J Immunol.

[B27] Li W, Murthy AK, Guentzel MN, Seshu J, Forsthuber TG, Zhong G, Arulanandam BP (2008). Antigen-specific CD4^+ ^T cells produce sufficient IFN-gamma to mediate robust protective immunity against genital *Chlamydia muridarum *infection. J Immunol.

[B28] Murthy AK, Cong Y, Murphey C, Guentzel MN, Forsthuber TG, Zhong G, Arulanandam BP (2006). Chlamydial protease-like activity factor induces protective immunity against genital chlamydial infection in transgenic mice that express the human HLA-DR4 allele. Infect Immun.

[B29] Williams DM, Grubbs BG, Pack E, Kelly K, Rank RG (1997). Humoral and cellular immunity in secondary infection due to murine *Chlamydia trachomatis*. Infect Immun.

[B30] Johansson M, Schon K, Ward M, Lycke N (1997). Genital tract infection with *Chlamydia trachomatis *fails to induce protective immunity in gamma interferon receptor-deficient mice despite a strong local immunoglobulin A response. Infect Immun.

[B31] Schall TJ, Bacon K, Toy KJ, Goeddel DV (1990). Selective attraction of monocytes and T lymphocytes of the memory phenotype by cytokine RANTES. Nature.

[B32] Kameyoshi Y, Dorschner A, Mallet AI, Christophers E, Schroder JM (1992). Cytokine RANTES released by thrombin-stimulated platelets is a potent attractant for human eosinophils. J Exp Med.

[B33] Taub DD, Oppenheim JJ (1994). Chemokines, inflammation and the immune system. Ther Immunol.

[B34] Palaniappan R, Singh S, Singh UP, Singh R, Ades EW, Briles DE, Hollingshead SK, Royal W, Sampson JS, Stiles JK, Taub DD, Lillard JW (2006). CCL5 modulates pneumococcal immunity and carriage. J Immunol.

[B35] Brunham RC, Rey-Ladino J (2005). Immunology of Chlamydia infection: implications for a *Chlamydia trachomatis* vaccine. Nature Rev Immunol Immunology.

[B36] Beagley KW, Eldridge JH, Kiyono H, Everson MP, Koopman WJ, Honjo T, McGhee JR (1988). Recombinant murine IL-5 induces high rate IgA synthesis in cycling IgA-positive Peyer's patch B cells. J Immunol.

[B37] Beagley KW, Eldridge JH, Lee F, Kiyono H, Everson MP, Koopman WJ, Hirano T, Kishimoto T, McGhee JR (1989). Interleukins and IgA synthesis. Human and murine interleukin-6 induce high rate IgA secretion in IgA-committed B cells. J Exp Med.

[B38] VanCott JL, Staats HF, Pascual DW, Roberts M, Chatfield SN, Yamamoto M, Coste M, Carter PB, Kiyono H, McGhee JR (1996). Regulation of mucosal and systemic antibody responses by T helper cell subsets, macrophages, and derived cytokines following oral immunization with live recombinant Salmonella. J Immunol.

[B39] Ifere GO, He Q, Igietseme JU, Ananaba GA, Lyn D, Lubitz W, Kellar KL, Black CM, Eko FO (2007). Immunogenicity and protection against genital *Chlamydia *infection and its complications by a multisubunit candidate vaccine. J Microbiol Immunol Infect.

[B40] Hickey DK, Bao S, Ikeda LT, Carey AJ, Beagley KW (2005). Induction of anti-chlamydial mucosal immunity by transcutaneous immunization is enhanced by topical application of GM-CSF. Curr Mol Med.

[B41] Singh UP, Singh S, Ravichandran P, Taub DD, Lillard JW (2004). Viral macrophage-inflammatory protein-II: a viral chemokine that differentially affects adaptive mucosal immunity compared with its mammalian counterparts. J Immunol.

[B42] Hickey DK, Jones RC, Bao S, Blake AE, Skelding KA, Berry LJ, Beagley KW (2004). Intranasal immunization with *C. muridarum *major outer membrane protein (MOMP) and cholera toxin elicits local production of neutralising IgA in the prostate. Vaccine.

[B43] Berry LJ, Hickey DK, Skelding KA, Bao S, Rendina AM, Hansbro PM, Gockel CM, Beagley KW (2004). Transcutaneous immunization with combined cholera toxin and CpG adjuvant protects against *Chlamydia muridarum *genital tract infection. Infect Immun.

[B44] Pal S, Luke CJ, Barbour AG, Peterson EM, de la Maza LM (2003). Immunization with the *Chlamydia trachomatis *major outer membrane protein, using the outer surface protein A of *Borrelia burgdorferi *as an adjuvant, can induce protection against a chlamydial genital challenge. Vaccine.

[B45] Su H, Parnell M, Caldwell HD (1995). Protective efficacy of a parenterally administered MOMP-derived synthetic oligopeptide vaccine in a murine model of *Chlamydia trachomatis *genital tract infection: serum neutralizing IgG antibodies do not protect against chlamydial genital tract infection. Vaccine.

[B46] Coffman RL, Varkila K, Scott P, Chatelain R (1991). Role of cytokines in the differentiation of CD4^+ ^T-cell subsets *in vivo*. Immunological Rev.

[B47] Chensue SW, Warmington KS, Allenspach EJ, Lu B, Gerard C, Kunkel SL, Lukacs NW (1999). Differential expression and cross-regulatory function of RANTES during mycobacterial (type 1) and schistosomal (type 2) antigen-elicited granulomatous inflammation. J Immunol.

[B48] An P, Nelson GW, Wang L, Donfield S, Goedert JJ, Phair J, Vlahov D, Buchbinder S, Farrar WL, Modi W, O'Brien SJ, Winkler CA (2002). Modulating influence on HIV/AIDS by interacting RANTES gene variants. Proc Natl Acad Sci USA.

[B49] Ramsey KH, Soderberg LS, Rank RG (1988). Resolution of chlamydial genital infection in B-cell-deficient mice and immunity to reinfection. Infect Immun.

[B50] Moore T, Ekworomadu CO, Eko FO, MacMillan L, Ramey K, Ananaba GA, Patrickson JW, Nagappan PR, Lyn D, Black CM, Igietseme JU (2003). Fc receptor-mediated antibody regulation of T cell immunity against intracellular pathogens. J Infect Dis.

